# Optical modulation of excitation-contraction coupling in human-induced pluripotent stem cell-derived cardiomyocytes

**DOI:** 10.1016/j.isci.2023.106121

**Published:** 2023-02-02

**Authors:** Vito Vurro, Beatrice Federici, Carlotta Ronchi, Chiara Florindi, Valentina Sesti, Silvia Crasto, Claudia Maniezzi, Camilla Galli, Maria Rosa Antognazza, Chiara Bertarelli, Elisa Di Pasquale, Guglielmo Lanzani, Francesco Lodola

**Affiliations:** 1 Center for Nano Science and Technology @PoliMi, Istituto Italiano di Tecnologia, Via Raffaele Rubattino, 81, 20134 Milan, Italy; 2 Department of Biotechnology and Biosciences, University of Milan-Bicocca, Building U3 - BIOS, Piazza della Scienza, 2, 20126 Milan, Italy; 3 Department of Chemistry, Materials and Engineering Chemistry ‘Giulio Natta’, Politecnico di Milano, Piazza Leonardo da Vinci, 32, 20133 Milan, Italy; 4 Institute of Genetic and Biomedical Research (IRGB), UOS of Milan, National Research Council of Italy, 20138 Milan, Italy; 5 IRCCS-Humanitas Research Hospital, 20089 Rozzano, Italy; 6 Department of Physics, Politecnico di Milano, Piazza Leonardo da Vinci, 32, 20133 Milan, Italy

**Keywords:** Molecular physiology, Cell biology, Stem cells research

## Abstract

Non-genetic photostimulation is a novel and rapidly growing multidisciplinary field that aims to induce light-sensitivity in living systems by exploiting exogeneous phototransducers. Here, we propose an intramembrane photoswitch, based on an azobenzene derivative (Ziapin2), for optical pacing of human-induced pluripotent stem cell-derived cardiomyocytes (hiPSC-CMs). The light-mediated stimulation process has been studied by applying several techniques to detect the effect on the cell properties. In particular, we recorded changes in membrane capacitance, in membrane potential (V_m_), and modulation of intracellular Ca^2+^ dynamics. Finally, cell contractility was analyzed using a custom MATLAB algorithm. Photostimulation of intramembrane Ziapin2 causes a transient V_m_ hyperpolarization followed by a delayed depolarization and action potential firing. The observed initial electrical modulation nicely correlates with changes in Ca^2+^ dynamics and contraction rate. This work represents the proof of principle that Ziapin2 can modulate electrical activity and contractility in hiPSC-CMs, opening up a future development in cardiac physiology.

## Highlights

• Ziapin2 partitions into the sarcolemma of hiPSC-CMs
• Upon photoexcitation, Ziapin2 modulates cell membrane capacitance
• This triggers action potential generation and the E-C coupling process
• Ziapin2-mediated effects open new perspectives for cardiac applications

## Graphical abstract

**Figure:**
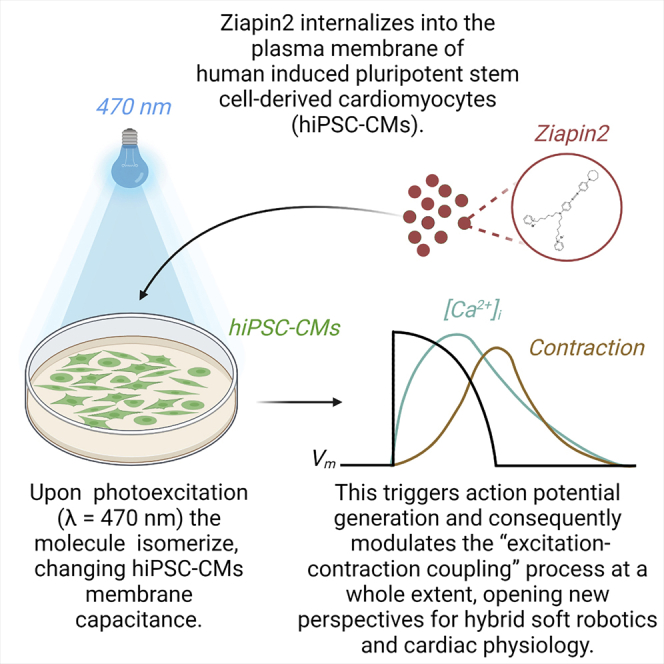

## Teaser

Molecular physiology; Nanotechnology; Stem cells research

## Introduction

In the cardiovascular field, optical stimulation is emerging as an alternative to traditional approaches for many research and therapeutic applications thanks to a series of key-enabling features: the lower energy consumption and release, and minimal invasiveness net of an extraordinary spatial and temporal resolution.^1^^,^^2^^,^^3^

Optogenetics,^4^^,^^5^^,^^6^^,^^7^^,^^8^^,^^9^ that has become widespread in neuroscience, could in principle be relevant in the cardiac field too.^10^^,^^11^^,^^12^^,^^13^^,^^14^^,^^15^^,^^16^^,^^17^^,^^18^^,^^19^^,^^20^^,^^21^^,^^22^ However, the approach still has a limited clinical applicability mainly because cell optical sensitivity is obtained by transduction with gene constructs carried by viral vectors.

A possible alternative strategy to overcome these constraints relies on the use of light-sensitive transducers, based on both inorganic and organic semiconductors.^23^^,^^24^^,^^25^^,^^26^^,^^27^^,^^28^^,^^29^^,^^30^^,^^31^^,^^32^ Those have been used recently as photoactive interfaces for cardiomyocyte (CM) optical stimulation. The generation of action potentials (APs) has been demonstrated by using planar graphene-based biointerfaces, possibly exploiting the photogeneration of charge carriers, and with polymer-silicon nanowires utilizing their photoelectrochemical properties.^25^^,^^30^ Similar results were obtained also increasing the local temperature with illumination of absorbers in contact with CMs, using gold nanoparticles or nanorod electrodes and metasurface planar organic interfaces.^31^^,^^33^^,^^34^ Organic semiconductors in particular allow to establish almost seamless abiotic/biotic interfaces, as they are soft materials that support both ionic and electronic transport, alike many biological molecules.^35^ In this context, the triggering mechanism leading to cell activity upon light absorption can be capacitive, faradaic, or thermal.^36^^,^^37^^,^^38^

An alternative approach, still based on organic molecules, exploits photochromic compounds that can be covalently bound^39^^,^^40^^,^^41^ to an ion channel or not-covalently^42^^,^^43^ to the plasma membrane. This methodology is gaining increasing interest due to the stimulation efficiency, its versatility, and the possibility of using two photon absorption that pushes the stimulation wavelengths to the near-infrared spectral region, improving tissue penetration.^44^^,^^45^

In this work, we propose a new tool for optical pacing of human-induced pluripotent stem cell-derived CMs (hiPSC-CMs) exploiting Ziapin2, a recently synthetized intramembrane photochromic transducer that we successfully tested in bacteria, non-excitable cells, and neurons.^46^^,^^47^^,^^48^^,^^49^^,^^50^

Ziapin2 has affinity for the hiPSC-CMs sarcolemma, and once partitioned, it undergoes *trans*-dimerization, which in turn leads to increased capacitance as the result of reduction in membrane thickness. Upon millisecond pulses of visible light, *trans*→*cis* isomerization causes a fast drop of capacitance due to membrane relaxation, resulting in a transient hyperpolarization followed by a delayed depolarization that triggers AP generation. The increase in AP frequency nicely correlates with changes in Ca^2+^ dynamics and contraction rate, thus proving that Ziapin2 modulates excitation-contraction (E-C) coupling at a whole extent.

## Results and discussion

### Ziapin2 photoisomerization modulates hiPSC-CMs membrane potential

The molecule was tested at two different concentrations (5 and 25 μM). In both cases, once added to hiPSC-CMs cultures, Ziapin2 successfully partitioned into the sarcolemma causing a significant increase in capacitance (Figure S1A). The effect was concentration dependent, as we observed a +23% rise (14.2 ± 1.2 pF vs 17.5 ± 0.9 pF; p = 0.05089) for 5 μM and +56% (14.2 ± 1.2 pF vs 22.2 ± 1.3 pF; p = 0.00063) for 25 μM, while no alterations were detected in hiPSC-CMs incubated with the vehicle (DMSO, data not shown). At equilibrium, this phenomenon changes the membrane electrical time constant (RC) but not the resting potential. According to previous experimental evidences,^46^^,^^48^^,^^49^ the modulation has been ascribed to the thinning of the bilayer caused by the dimerization of Ziapin2 molecules inside the membrane.

Upon photostimulation, the azobenzene isomerization removes the geometric constraint of the dimer leading to membrane relaxation and a partial return toward steady-state capacitance values with a reduction of −14% (17.5 ± 0.9 pF vs 15.0 ± 0.7 pF; p = 0.08) and −12% (22.2 ± 1.3 pF vs 19.5 ± 1.1 pF; p = 0.14) for 5 and 25 μM Ziapin2, respectively (Figure S1B).

**Figure 1:**
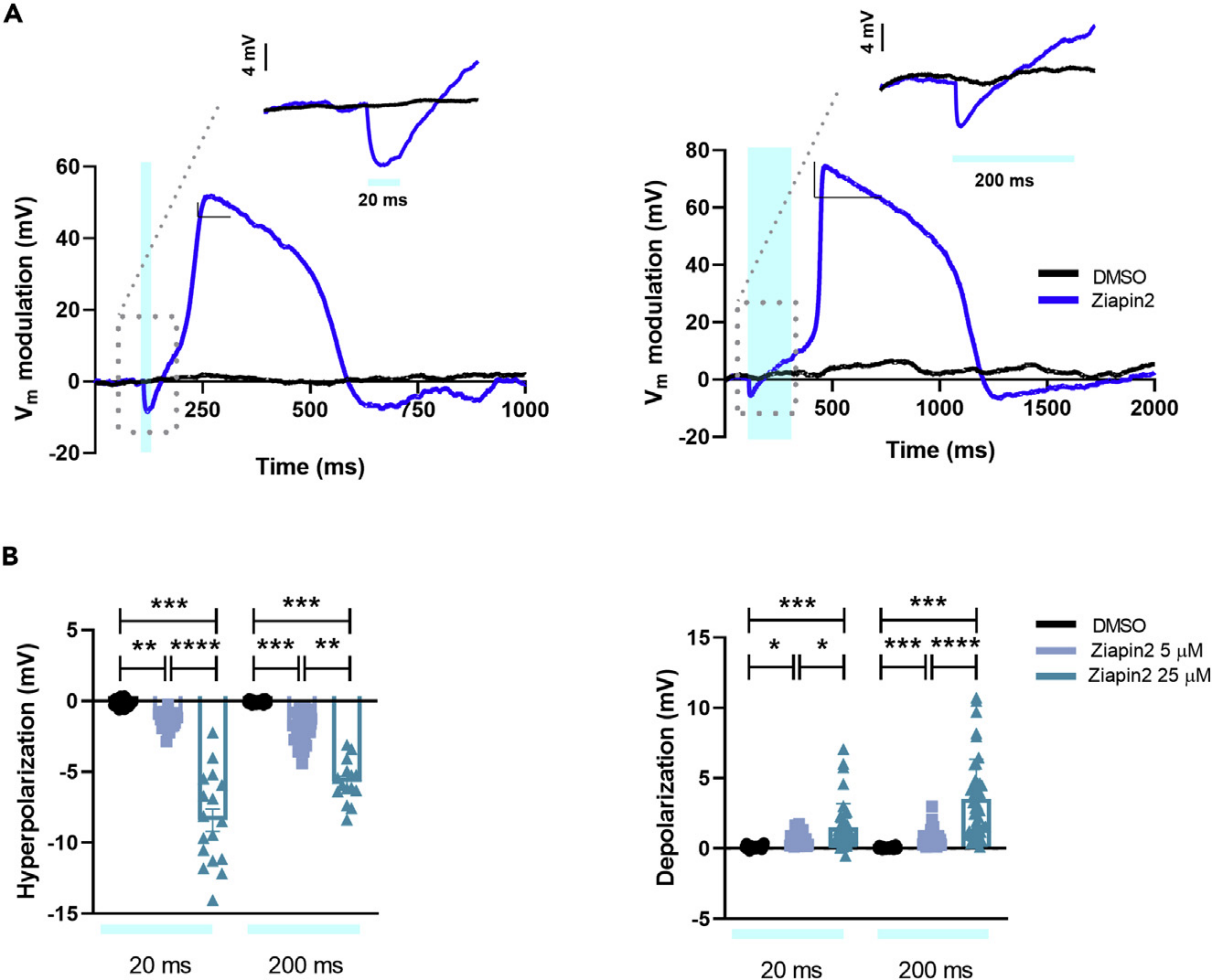
Ziapin2 mediates a light-evoked membrane voltage modulation in hiPSC-CMs (A) Representative whole-cell current-clamp traces recorded in cells loaded with either vehicle (DMSO, in black) or Ziapin2 (blue) and stimulated with 20 ms- (left) or 200 ms-long (right) single light pulses. Photoexcitation is represented by the cyan shaded area. Light power density, 80 mW/mm^2^. The membrane potential modulation can be appreciated at a faster timescale in the inset of each panel. (B) Scatterplots of the peak hyperpolarization (left) and depolarization (right) changes in hiPSC-CMs exposed to Ziapin2 (5 μM in sky blue, 25 μM in teal) or DMSO for the above-mentioned light-stimulation protocols. n > 18 for 5 μM and 25 μM Ziapin2-loaded cells and n = 20 for DMSO-treated hiPSC-CMs. V_m_ values have been reported as relative variation to better appreciate the light-induced effect; however, no significant changes were detected in the resting membrane potential values (Ziapin2 5 μM Vm = -42.4 ± 3.9 mV; Ziapin2 25 μM Vm = -42.8 ± 2.6 mV; DMSO Vm = -37 ± 4.4 mV). The experiments were carried out at room temperature (24°C). Data were collected from three independent differentiations and are represented as mean ± SEM ∗p < 0.05, ∗∗p < 0.01, ∗∗∗p < 0.001 and ∗∗∗∗p < 0.0001.

Whole-cell patch clamp experiments in current clamp mode (I = 0) revealed that the photoinduced drop in capacitance correlates with a modulation of the membrane potential. In particular, the Ziapin2-mediated photostimulation (with 20 and 200 ms single light pulses) results in a transient hyperpolarization that is occurring within few ms after the light onset, consistent with the linear inverse relation between capacitance and voltage. The recovery of the potential by capacitive currents is followed by a depolarization which is often large enough to generate an AP (see representative traces in Figure 1A).

Both hyperpolarization and depolarization peaks display much higher amplitude at 25 μM concentration than 5 μM (Figure 1B). For instance, with 20 ms light stimulation, hyperpolarization is six times larger at 25 μM concentration, even if the relative change in capacitance is comparable (−14% vs −12% for 5 and 25 μM Ziapin2, respectively, see Figure S1B). This is surprising, since according to the simple plane capacitor model, if there are no other changes in the circuit, ΔV/V = ΔC/C. To explain this discrepancy, we conjecture that equal amplitude, but faster changes in capacitance, could give rise to larger changes in membrane potential. This is confirmed by a numerical simulation based on the equivalent circuit model for the membrane (Figure S2). To account for the different kinetics, we propose that at higher concentration, Ziapin2 dwells in a more heterogeneous membrane environment, characterized by different viscosity, order, and phospholipid polarity, where a faster isomerization might occur.

In agreement with capacitance data, no light-dependent effects were noticed in vehicle-treated hiPSC-CMs (Figure 1).

### Ziapin2 photoisomerization induces AP firing in hiPSC-CMs

**Figure 2:**
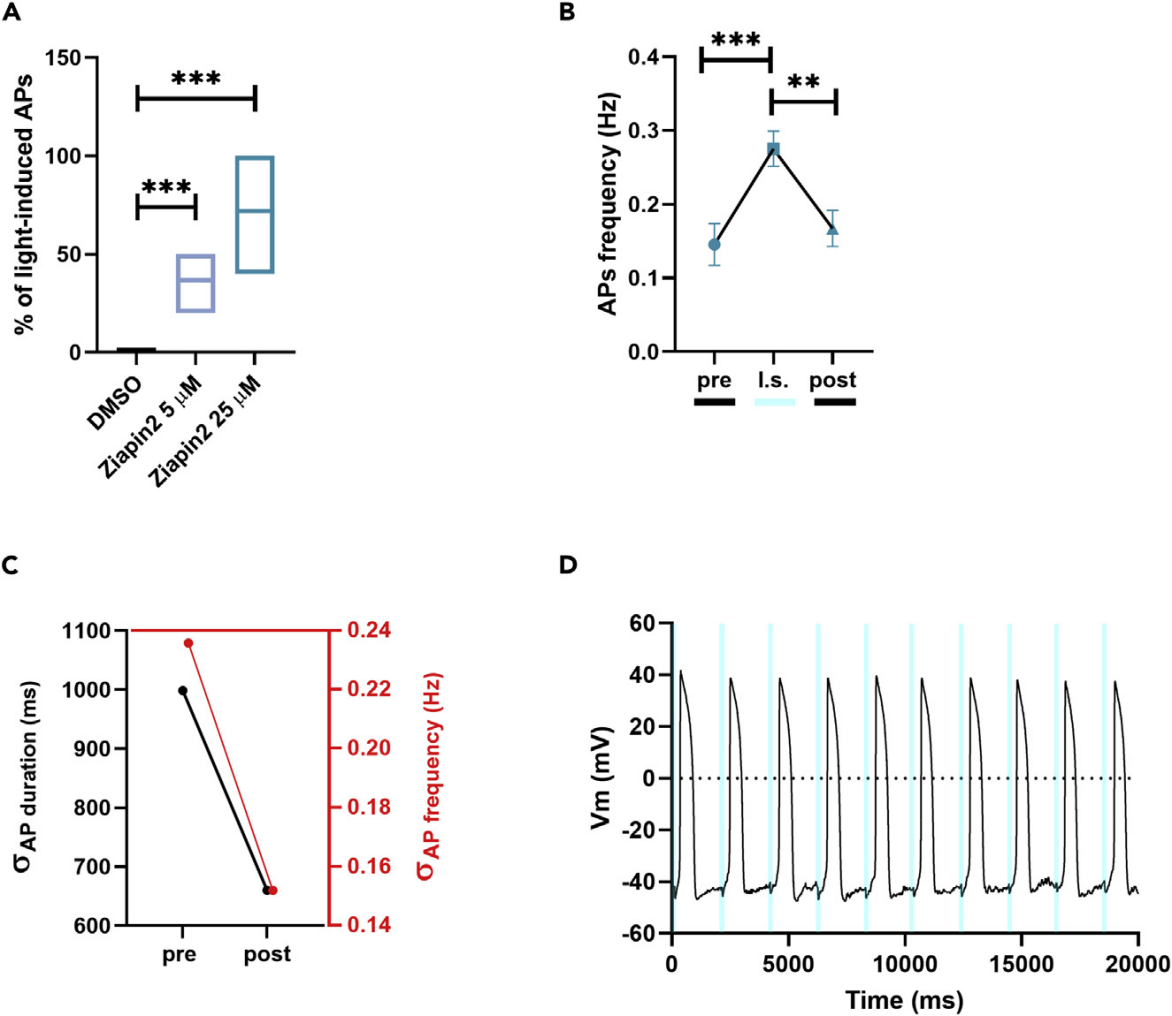
Light-evoked AP modulation by Ziapin2 in hiPSC-CMs (A) Boxplots (line at mean) of the reproducibility of the light-induced AP generation in cells loaded with either vehicle (DMSO, in black) or Ziapin2 (5 μM, in sky blue; 25 μM, in teal). The percentage represents the n° of AP directly generated by each light pulse. (B) AP frequency before (pre), during (l.s.), and after (post) light stimulation. Data are represented as mean ± SEM; n > 40 for each condition from three independent differentiations. (C) Correlation between the SD(σ) of AP duration (left, black) and frequency (right, red) pre- and post-photostimulation. (D) Representative traces of APs generated in response to a light train stimulation (20 ms cycle length; 0.5 Hz stimulation frequency, represented as cyan shaded areas). Light power density, 80 mW/mm^2^. The experiments were carried out at room temperature (24°C). ∗∗p < 0.01 and ∗∗∗p < 0.001.

To assess the impact of the molecule on hiPSC-CMs electrical activity, we evaluated the ability of light stimulation to generate APs in hiPSC-CMs loaded with Ziapin2. Remarkably, the most striking effect was observed in CMs exposed to the highest photochromic concentration (72 ± 4% of responding cells for Ziapin2 25 μM vs 37 ± 3% for Ziapin2 5 μM, Figure 2A).

As a next step, we compared spontaneous and light-evoked AP features. Since the most significant effects occurred in hiPSC-CMs loaded with Ziapin2 25 μM and subjected to 20 ms light stimuli, here we report only data within these experimental conditions.

In dark conditions, no significant differences were measured in maximum diastolic potential (Figure S3A) and maximal upstroke velocity (dV/dt_max_, Figure S3B), while AP amplitude (APA, Figure S3C) was slightly decreased in Ziapin2-exposed CMs (70.3 ± 1.2 mV vs 70.8 ± 2.9 mV; p = 0.04). Ziapin2 photoisomerization did not exert any detectable modification of these AP parameters (Figures S3B and S3C), while a light-triggered increase in the number of AP per unit time was observed (0.14 ± 0.02 Hz vs 0.27 ± 0.02 Hz for spontaneous and light-evoked, respectively; p < 0.0001, Figure 2B). This light-induced frequency increment was associated with a shortening of the AP duration; plotting the SD of these two parameters, it was possible to notice a greater clustering of both the indicators upon illumination (Figure 2C). This suggests that Ziapin2 can potentially play a pivotal role in making homogeneous activity of cardiac cells and tissues by reducing the overall beating variabilities. Interestingly, a punctual AP generation was obtained when a stimulation train of 20 ms light pulses at 0.5 Hz was applied, observing just few failures under rate-controlled conditions (Figure 2D). We attributed the missed stimulations to the low temperature (24°C) used in our experiments. It is indeed known that a more physiological temperature would speed up the dynamics of cellular processes. Accordingly, we monitored the electrical activity of hiPSC-CMs at 37°C (Figure S4). As expected, in these conditions, there was a higher spontaneous electrical activity (∼0.78 Hz, Figure S4A, left panel) that increased upon Ziapin2-mediated photostimulation (∼0.95 Hz, Figure S4B, left panel). Notably, considering a sampling time of 2 s, we observed that the APs under control conditions are randomly distributed (Figure S4A, right panel), while upon photoexcitation they cluster at the light onset (Figure S4B, right panel). This means that the spontaneous APs are generated in deterministic way and that we are imposing a 0.5 Hz stimulation frequency.

From this small subset of data, we also extrapolate the AP duration at 90% of repolarization (APD_90_, directly correlated with the frequency increase) and APA. In Figure S4C are reported data acquired at both 24°C and 37°C to show that, as expected, the increase in temperature mainly affects APD_90_, leaving the amplitude unaltered.

### Ziapin2 photoisomerization elicits Ca^2+^ dynamics in hiPSC-CMs

**Figure 3:**
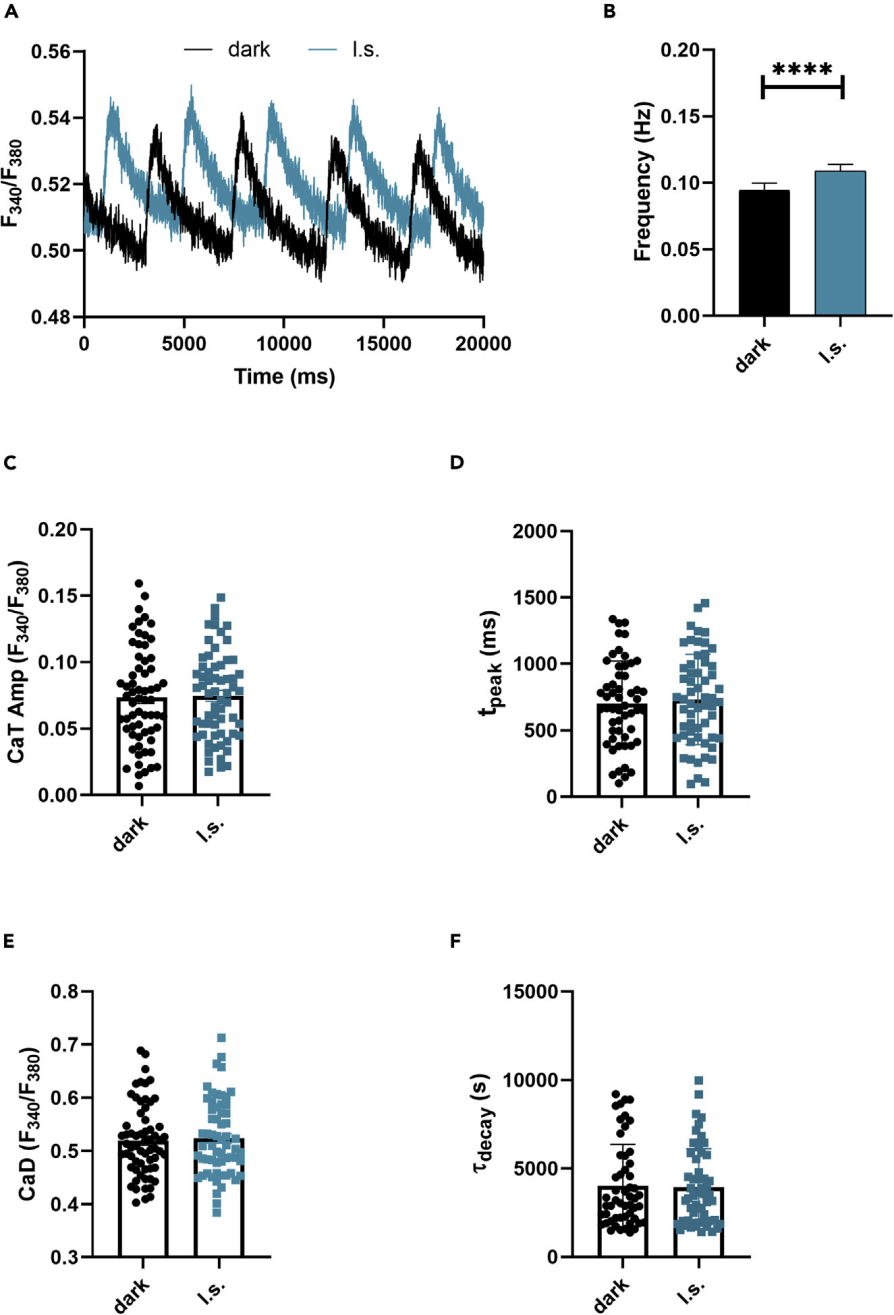
Photoinduced Ca^2+^ transients in Ziapin2-loaded hiPSC-CMs *(*A) Representative Ca^2+^ transients (CaTs) recorded on the same Ziapin2-loaded cell first in dark condition (dark, in black) and subsequently photostimulated with a 200 ms pulse (l.s., in teal) at light power density = 273 μW/mm^2^. (B) Frequency of CaTs, (C) CaT amplitude (CaT Amp), (D) CaT rise-time (t_peak_), (E) Diastolic Ca^2+^ (CaD), and (F) CaT decay kinetics (τ_decay_). The paired measurements were carried out at room temperature (24°C) on 63 cells. Data are represented as mean ± SEM. ∗∗∗∗p < 0.0001.

In CMs, the AP triggers the E-C coupling, namely the physiological process that converts an electrical stimulus to a mechanical response. It is well established that the contraction of cardiac cells is triggered by a considerable increase in intracellular Ca^2+^ concentration. We thus evaluated Ziapin2 effect on cytoplasmic Ca^2+^ levels performing fluorescence-based measurements with the widely used ratiometric Ca^2+^ indicator Fura-2 AM (Figure 3A).

To monitor if the molecule photoisomerization had an impact on Ca^2+^ dynamics, we first measured the spontaneous Ca^2+^ activity by keeping Ziapin2-loaded hiPSC-CMs in dark conditions, and subsequently, we subjected the same cells to a single light stimulation pulse using an arc lamp light source coupled with a high-speed OptoScan monochromator that allowed us to accurately select the excitation wavelength.

In accordance with the patch-clamp data, we observed a significantly higher frequency of Ca^2+^ transients (CaTs) (+15.4%, p = 0.0001, Figure 3B) when hiPSC-CMs were subjected to light stimulation while no significant changes were detected in the amplitude (p = 0.6, Figure 3C), in the rise-time (p = 0.93, Figure 3D), or in the decay kinetics (p = 0.67, Figure 3E) of the CaTs. Moreover, the diastolic Ca^2+^ levels were also comparable among the two groups (Figure 3F).

Since changes in the shape of the CaT might reflect alterations in cardiac ion channels or cardiac signaling pathways, we can speculate that is unlikely that a primary membrane transporter like Na^+^/Ca^2+^ exchanger (NCX) or the plasma membrane Ca^2+^ ATPase is altered upon Ziapin2 photoisomerization, suggesting that the molecule acts by fully respecting the physiological behavior of the cell.

### Ziapin2 photoisomerization enhances hiPSC-CMs contraction rate

**Figure 4:**
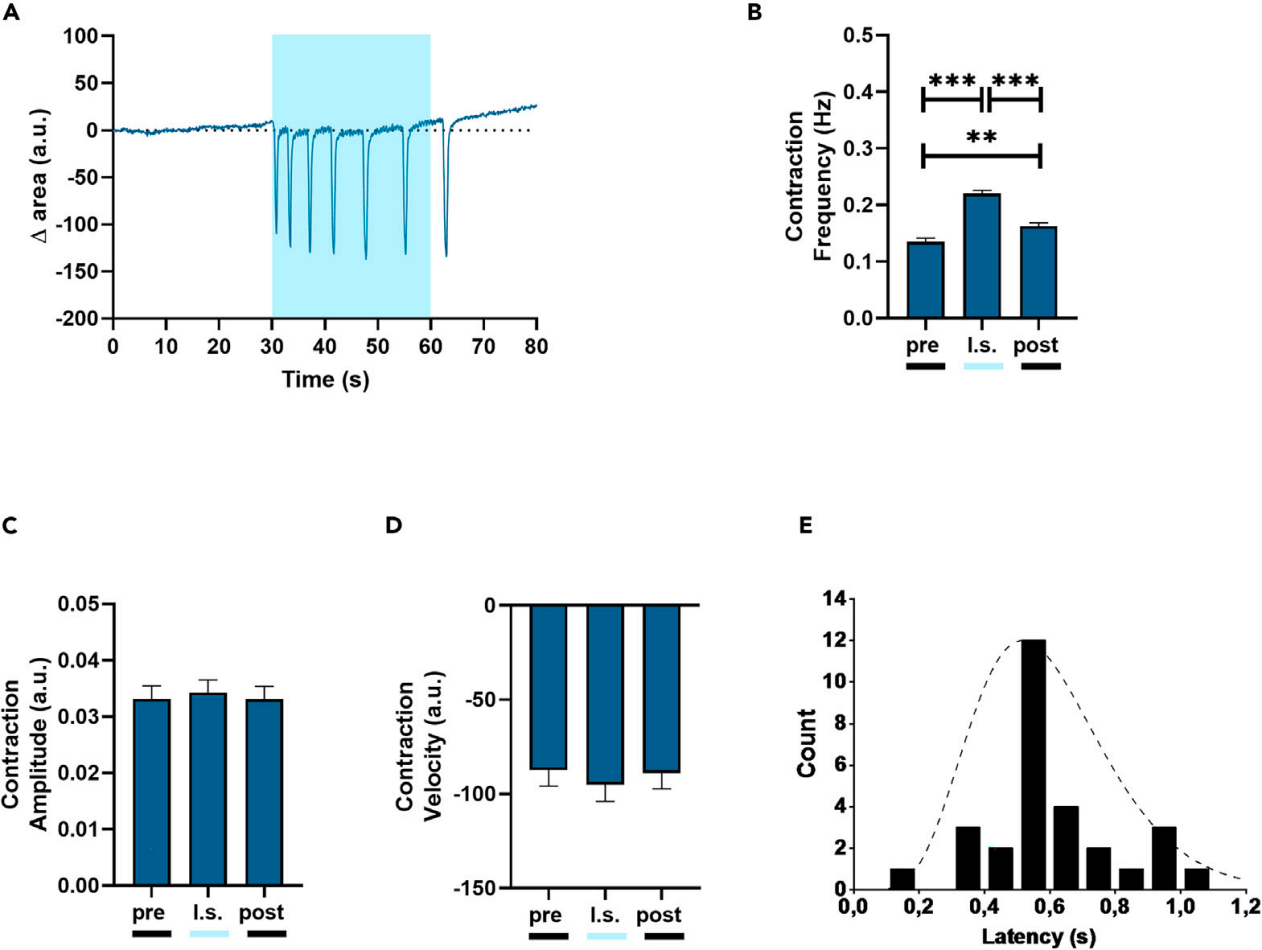
Light-induced contraction rate modulation in Ziapin2-loaded hiPSC-CMs (A) Representative trace of the contraction behavior of Ziapin2-treated hiPSC-CMs. (B) Contraction frequency, (C) Contraction amplitude, and (D) Contraction velocity analyzed before (pre), during (l.s.), and after (post) 1 Hz pulsed light stimulation at light power density = 30 mW/mm^2^ (n = 220 from three independent differentiations, data are shown as mean ± SEM). (E) Time to contraction measured from the light onset in quiescent hiPSC-CMs (n = 29). The cell response time distribution peaks at 500 ms, highlighting a correlation between light stimulation and cell’s functional response. A gamma function has been used as a guide to the eye of this time distribution. The experiments were carried out at room temperature (24°C). ∗∗p < 0.01 and ∗∗∗p < 0.001.

As a final step, we investigated the effect of Ziapin2 on hiPSC-CMs contraction behavior by exploiting a custom MATLAB code that allowed us to obtain a quantitative analysis from previously acquired high-speed movies (Figure 4).

In dark condition, Ziapin2-loaded hiPSC-CMs had a contraction rate of 0.13 ± 0.006 Hz. Under 1 Hz pulsed illumination, the rate increased by 59% during the entire acquisition window (Figure 4B), while it remained constant in vehicle-treated hiPSC-CMs subjected to the same stimulation protocol (Figure S5). The augmented frequency occurs net of variations in contraction amplitude (calculated as area reduction, p = 0.74, Figure 4C) and contraction velocity (extracted as derivative of the contraction amplitude, p = 0.57, Figure 4D). Interestingly, the effect on the contraction frequency persisted in the 25–30 s following light offset, albeit with a 27% decrease (from 0.22 ± 0.006 Hz to 0.16 ± 0.0059 Hz, Figure 4B). There are two possible explanations for this; first, the experiments were performed at 24°C, a temperature far from the canonical physiological 37°C that could have slowed down the cell behavior.^29^ Secondly, even though the hiPSC-CMs are a consolidated tool and hold an enormous potential in cardiovascular research, their E-C coupling process resembles more the one of fetal/neonatal CMs, thus it is possible that the Ca^2+^ clearance machinery in these cells is substantially slower in comparison to adult CMs.^51^^,^^52^

Remarkably, the lower the spontaneous hiPSC-CMs contraction rate, the higher the light-induced frequency increase. To better characterize the involvement of Ziapin2 in the light-induced increase in contraction frequency, we measured the latency, calculated as the interval between the onset of stimulation and the contraction of the CMs, in a small subset of quiescent cells (Figure 4E).

The timescale was found to be distributed around 500 ms in a non-stochastic way, compatibly, at least in these experimental conditions, with expected times for the electromechanical coupling, thus confirming the link between optical stimulus and increased contraction.

### Conclusions & perspectives

The possibility to manipulate the cellular activity with a precise spatial and temporal punctuality is a key enabling technology for biology and medicine. In this work, we report the first proof-of-principle demonstration of Ziapin2 as a light-sensitive tool to control cardiac cell excitability and contractility. Our approach is innovative for a series of reasons: i) unlike electrical stimulation, it enables a non-contact and reversible excitation with high spatiotemporal precision; ii) it avoids genetic manipulation and the pitfalls related to the introduction of exogenous genetic material using viral vectors, thus overcoming the major drawback of optogenetics; iii) Ziapin2 primarily targets the cellular passive properties without affecting ionic conductances, an issue shared by both optogenetics and photoswitchable ligands, still allowing a millisecond control of V_m_; iv) the molecule effect is heatless, thus preventing increases in temperature that could be potentially harmful to the cells over the long term.

Ziapin2 could be used as a non-invasive optical tool to control cardiac electrical activity for bio-hybrid robotics studies (in which CMs are often the biological substrate), thus enabling or simplifying design and fabrication of the actuators.^53^^,^^54^^,^^55^ Also, the possibility to pattern light in cardiac organoids or microtissues will allow to address fundamental questions of cardiac biology.

### Limitations of the study

Further studies are needed to elucidate the biophysical mechanisms ruling the photostimulation process. In particular, future research will be devoted to the study of the triggering mechanism that leads to AP generation and to exclude the potential involvement of membrane transporters like NCX, Na^+^ or K^+^-ATPase, as well as membrane receptors (e.g., β-adrenergic receptors). Moreover, some chemical improvement should be faced to increase the molecular absorption cross section leading to a reducing of the required light power density. Similarly, to further corroborate our findings, we will extend this approach to fully differentiated adult cardiomyocytes, either human- or mouse-derived. This will be an important step to show that our molecules do work in a more complex and physiological scenario.

If successful, this could open a broader field of applications for light-mediated clinical treatment of cardiac arrhythmias. In this context, new curative strategies that are less invasive, long-lasting, and less power consuming than the existing ones are a *conditio sine qua non* to reduce the burden for the recipients.

## STAR★Methods

### Key resources table

**Table:** 

REAGENT or RESOURCE	SOURCE	IDENTIFIER
**Biological samples**		

hiPSC-CMs	Ncardia	Pluricyte® Cardiomyocyte, PCMI-1033-1
hiPSC-CMs	Humanitas Clinical and Research Center-IRCCS	See “Experimental Model and subject details” section for the differentiation protocol.

**Chemicals, peptides, and recombinant proteins**		

Fura-2-AM	Thermofisher Scientific	F1201
hiPSC growth medium	Thermofisher Scientific	StemFlex Medium (A3349401)
Vitronectin (VTN-N) Recombinant Human Protein, Truncated	Thermofisher Scientific	A14700
hiPSC-CMs medium	Ncardia	Pluricyte® Cardiomyocyte Medium, PM-2100-100mL
B27-Supplement w/o insulin	Thermofisher Scientific	0050129SA
B27-Supplement	Thermofisher Scientific	17504044
Growth Factor-Reduced Matrigel (10ml)	BD Biosciences	354230
RPMI Medium 1640 with L-Glutamine	Thermofisher Scientific	11875-119
CHIR-99021	Selleckchem	S1263-5MG
IWR1	Sigma Aldrich	10161-5MG
Y-27632 (ROCK Inhibitor)	Selleckchem	S1049
Laminin	Sigma Aldrich	L2020
Fibronectin	Sigma Aldrich	F0895-2MG

**Software and algorithms**		

Microsoft Excel	Microsoft	https://www.microsoft.com/
GraphPad Prism	Prism	https://www.graphpad.com/
MATLAB	MathWorks	https://it.mathworks.com/
Origin 9.0	OriginLab Corporation	https://www.originlab.com/
pClamp-10 software	Axon Instruments	http://go.moleculardevices.com/
Contraction analysis code	This paper	[GitHub]: [https://github.com/BeatriceFederici/contraction_tracking.git]

**Other**		

Patch-Clamp Amplifier	Molecular Devices	Axopatch 200B
Patch-Clamp Amplifier	Molecular Devices	MultiClamp 700B
Analog to digital converter	Molecular Devices	Digidata 1440A
Microscope	Nikon	Eclipse Ti
Microscope	Nikon	Eclipse TE200
Illumination source	Lumencor	Spectra X
Illumination source	Cairn Research	Arc Lamp Light Source coupled with high-speed OptoScan monochromator
Camera	Photometrics	Prime 95B Scientific CMOS (sCMOS) Camera
Camera	ThorLabs	CS135MUN, Kiralux 1.3 MP NIR-Enhanced CMOS Camera

### Resource availability

#### Lead contact

Further information and requests for resources and reagents should be directed to and will be fulfilled by the lead contact, Francesco Lodola (francesco.lodola@unimib.it).

#### Materials availability

This study did not generate any unique new reagents.

### Experimental model and subject details

#### Cell culturing and differentiation

Two different batches of hiPSC-CMs were used for this study, a commercial one (Pluricyte® CMs provided by Ncardia, Gosselies, Belgium) and a laboratory handle one. The latter was produced by directed cardiac differentiation of previously generated control hiPSCs.^29^^,^^56^^,^^57^

In brief, cardiac induction and generation of hiPSC-CMs were obtained as previously reported, using a chemically-defined serum-free protocol, which is based on activation (CHIR99021) and inhibition (IWR1) of the Wnt pathway in RPMI-B27 medium.^58^^,^^59^^,^^60^ In this work, CMs were differentiated from two different control iPSC lines (one male and one female) between the 18^th^ and the 35^th^ passage in culture. Importantly, employed cell lines were regularly tested for being free of major chromosomal abnormalities by karyotype analysis. CMs were used for the experiments 25–30 days after spontaneous contracting activity was started, a differentiation stage at which they expressed the repertoire of sarcomeric proteins, calcium regulators, and ionic channels necessary for their correct functionality. Purity of differentiated CM populations was regularly checked before each experiment to be greater than 90% (not shown). The commercial batch was thawed and cultured following the manufacturer’s protocol. In terms of plating density, cells for both batches were seeded onto the material at a confluence comprised between 50% and 60% on fibronectin coated (15 μg/ml in PBS buffer solution) 18-mm round glass coverslip (VWR, Radnor, USA). The cells were maintained in incubator at 37°C and 5% CO_2_.

### Method details

#### Ziapin2 synthesis and uptake process

Ziapin2 was synthetized as previously described.^46^^,^^48^^,^^49^ Micromolar concentrations (5 or 25 μM) of the compound were added to hiPSC-CMs cultures directly into the culture medium. Subsequently the cells were placed in the incubator at 37°C and 5% of CO_2_. After 7 minutes the medium was gently washed out and the petri dish was rinsed with fresh extracellular solution.

#### Electrophysiology

Standard whole-cell patch clamp recordings were performed on isolated cells with an Axopatch 200B (Axon Instruments) coupled with a Nikon Eclipse Ti inverted microscope. Acquisitions were performed with freshly pulled glass pipettes (4-7 MΩ), filled with the following intracellular solution [mM]: 12 KCl, 125 K-Gluconate, 1 MgCl_2_, 0.1 CaCl_2_, 10 EGTA, 10 HEPES, 10 ATP-Na_2_. The extracellular solution contained [mM]: 135 NaCl, 5.4 KCl, 5 HEPES, 10 Glucose, 1.8 CaCl_2_, 1 MgCl_2_. Acquisitions were performed with pClamp-10 software (Axon Instruments). Membrane currents were low pass filtered at 2 kHz and digitized with a sampling rate of 10 kHz (Digidata 1440 A, Molecular Devices).

A cyan excitation light (λ_ex_ = 470 nm) was provided by a LED system (Lumencor Spectra X) coupled to the fluorescence port of the microscope through a liquid-fibre. The photoexcitation density was 80 mW/mm^2^, as measured at the output of the 40X microscope objective (Pobj). Hyperpolarization and depolarization were measured as the minimum and maximum voltage, respectively, reached within 350 ms from the light-onset. Data were analyzed with Origin 9.0 (OriginLab Corporation) and MATLAB (MathWorks).

#### Capacitance recordings

Capacitance measurements were performed as previously described.^49^^,^^61^ Briefly, a double sinusoidal voltage clamp signal was applied to the cell in whole-cell configuration. The response current signal was acquired, and membrane capacitance and resistance were extracted fitting the current with a custom MATLAB program. The capacitance value was extracted in dark condition and during light stimulation (20 ms pulse). The shorter pulse was used in order to consider mainly the effect related to Ziapin2 photoisomerization.

#### Intracellular Ca^2+^ measurements

Intracellular Ca^2+^ measurements were performed with a Nikon Eclipse TE200 microscope, and the extracellular solution was the one used for the electrophysiology measurements. Ca^2+^ dynamics were recorded in hiPSC-CMs incubated with 2 μM Fura-2 AM probe (Ex: 340/380 nm; Em: 510 nm) for 30 minutes and washed out for 5 minutes with extracellular solution. Ziapin2 (470 nm at power density of 273 μW/mm^2^) and Fura-2 AM excitations (340 and 380 nm, at power density 63 and 173 μW/mm^2^, respectively) were provided using the same arc lamp light source coupled with high-speed OptoScan monochromator that allowed us to accurately select the wavelength. To collect local signals only (kinetics not distorted by propagation), a diaphragm in the optical path was adjusted to delimit a region of interest (ROI) enclosing single cells. The intracellular concentration of Ca^2+^ ([Ca^2+^]_i_) was monitored by measuring, for each ROI, the ratio of the mean fluorescence emitted at 510 nm when exciting alternatively at 340 and 380 nm (shortly termed ‘‘ratio’’), four to five ROIs were analyzed in each plate. An increase in [Ca^2+^]_i_ causes an increase in the ratio. The following parameters were extracted from each CaT: diastolic Ca^2+^ (CaD), CaT amplitude (CaT Amp), CaT rise-time (t_peak_) and CaT decay kinetics (τ_decay_).

#### Acquisition and analysis of hiPSC-CMs contractile behavior

The Nikon Eclipse Ti inverted microscope described before was used to stimulate (Lumencor Spectra X, λ_ex_ = 470 nm, 20X objective, Pobj = 30 mW/mm^2^) and acquire video of the CMs contraction a frequency. An objective with a lower magnification was used to increase the amount of recorder cells resulting in a more robust analysis. Consequently, the resulting power density was lower due to the bigger area.

Contractile behavior was analyzed using a custom-built algorithm, implemented in MATLAB R2020a. The approach is based on the contraction-induced retraction of cell body towards nucleus and is effective even if there is no displacement of cell extremities or if the cellular edge cannot be detected properly.

The user defines one or more regions of interest (ROI) using bounding boxes. Each bounding box should contain one single cell. For each ROI a set of features is identified and tracked across video frames by means of Kanade-Lucas-Tomasi algorithm.^62^^,^^63^ Since the ROI is delimiting a cell, the tracked features are expected to belong to cell body. Averaging the estimated motion fields of all these feature points returns a mean geometric transformation, which can be applied to the bounding box delimiting the ROI. The area of this bounding box is measured over time and presents minima at cellular contractions. The number of minima per time interval yields an estimate of the cell contraction rate. Contraction amplitude is measured as percent reduction of bounding box area at contraction and averaged over all contractions within a specific running observation window (e.g., pre). The box area derivative when cells contract evaluates cell contraction velocity. Data were analyzed with Origin 9.0 (OriginLab Corporation).

### Quantification and statistical analysis

Data were expressed as mean ± SEM. Normal distribution was assessed using D’Agostino-Pearson’s normality test. To compare two sample groups, either the Student’s t-test or the Mann-Whitney U-test was used. To compare more than two sample groups, one-way ANOVA or Kruskal-Wallis were used. p < 0.05 was considered statistically significant. The analysis was carried out using Origin (OriginLab) and Prism (GraphPad).

## Data Availability

• All the data reported in this paper will be shared by the lead contact upon request. • All original code has been deposited to Github and is publicly available as of the date of publication. The DOI is listed in the key resources table. • Any additional information required to re-analyze the data reported in this paper is available from the lead contact upon request.

## References

[1] Di Maria F., Lodola F., Zucchetti E., Benfenati F., Lanzani G. (2018). The evolution of artificial light actuators in living systems: from planar to nanostructured interfaces. Chem. Soc. Rev..

[2] Antognazza M.R., Martino N., Ghezzi D., Feyen P., Colombo E., Endeman D., Benfenati F., Lanzani G. (2015). Shedding light on living cells. Adv. Mater..

[3] Hopkins J., Travaglini L., Lauto A., Cramer T., Fraboni B., Seidel J., Mawad D. (2019). Photoactive organic substrates for cell stimulation: progress and perspectives. Adv. Mater. Technol..

[4] Deisseroth K. (2011). Nat. Methods.

[5] Fenno L., Yizhar O., Deisseroth K. (2011). The development and application of optogenetics. Annu. Rev. Neurosci..

[6] Rein M.L., Deussing J.M. (2012). The optogenetic (r)evolution. Mol. Genet. Genomics..

[7] Deisseroth K. (2015). Optogenetics: 10 years of microbial opsins in neuroscience. Nat. Neurosci..

[8] Duebel J., Marazova K., Sahel J.-A. (2015). Optogenetics. Curr. Opin. Ophthalmol..

[9] Dwenger M., Kowalski W.J., Ye F., Yuan F., Tinney J.P., Setozaki S., Nakane T., Masumoto H., Campbell P., Guido W., Keller B.B. (2019). Chronic optical pacing conditioning of h-iPSC engineered cardiac tissues. J. Tissue Eng..

[10] Knollmann B.C. (2010). Pacing lightly: optogenetics gets to the heart. Nat. Methods.

[11] Bruegmann T., Malan D., Hesse M., Beiert T., Fuegemann C.J., Fleischmann B.K., Sasse P. (2010). Optogenetic control of heart muscle in vitro and in vivo. Nat. Methods.

[12] Ambrosi C.M., Klimas A., Yu J., Entcheva E. (2014). Cardiac applications of optogenetics. Prog. Biophys. Mol. Biol..

[13] Boyle P.M., Karathanos T.V., Trayanova N.A. (2018). Cardiac optogenetics: 2018. JACC. Clin. Electrophysiol..

[14] Asano T., Igarashi H., Ishizuka T., Yawo H. (2018). Organelle optogenetics: direct manipulation of intracellular Ca2+ dynamics by light. Front. Neurosci..

[15] Ferenczi E.A., Tan X., Huang C.L.-H. (2019). Principles of optogenetic methods and their application to cardiac experimental systems. Front. Physiol..

[16] Sasse P., Funken M., Beiert T., Bruegmann T. (2019). Optogenetic termination of cardiac arrhythmia: mechanistic enlightenment and therapeutic application?. Front. Physiol..

[17] Krueger D., Izquierdo E., Viswanathan R., Hartmann J., Pallares Cartes C., De Renzis S. (2019). Principles and applications of optogenetics in developmental biology. Development.

[18] Huang C.L.-H., Ferenczi E.A., Lei M. (2020). Editorial: optogenetics: an emerging approach in cardiac electrophysiology. Front. Physiol..

[19] Floria M., Radu S., Gosav E.M., Moraru A.C., Serban T., Carauleanu A., Costea C.F., Ouatu A., Ciocoiu M., Tanase D.M. (2020). Cardiac optogenetics in atrial fibrillation: current challenges and future opportunities. BioMed Res. Int..

[20] Li J., Li H., Rao P., Luo J., Wang X., Wang L. (2021). Shining light on cardiac electrophysiology: from detection to intervention, from basic research to translational applications. Life Sci..

[21] Crocini C., Ferrantini C., Coppini R., Scardigli M., Yan P., Loew L.M., Smith G., Cerbai E., Poggesi C., Pavone F.S., Sacconi L. (2016). Optogenetics design of mechanistically-based stimulation patterns for cardiac defibrillation. Sci. Rep..

[22] Biasci V., Santini L., Marchal G.A., Hussaini S., Ferrantini C., Coppini R., Loew L.M., Luther S., Campione M., Poggesi C. (2022). Optogenetic manipulation of cardiac electrical dynamics using sub-threshold illumination: dissecting the role of cardiac alternans in terminating rapid rhythms. Basic Res. Cardiol..

[23] Starovoytov A., Choi J., Seung H.S. (2005). Light-directed electrical stimulation of neurons cultured on silicon wafers. J. Neurophysiol..

[24] Bareket-Keren L., Hanein Y. (2014). Novel interfaces for light directed neuronal stimulation: advances and challenges. Int. J. Nanomedicine.

[25] Savchenko A., Cherkas V., Liu C., Braun G.B., Kleschevnikov A., Miller Y.I., Molokanova E. (2018). Graphene biointerfaces for optical stimulation of cells. Sci. Adv..

[26] Rand D., Jakešová M., Lubin G., Vėbraitė I., David-Pur M., Đerek V., Cramer T., Sariciftci N.S., Hanein Y., Głowacki E.D. (2018). Direct electrical neurostimulation with organic pigment photocapacitors. Adv. Mater..

[27] Feiner R., Dvir T. (2019). A ray of light for treating cardiac conduction disorders. Proc. Natl. Acad. Sci. USA..

[28] Lodola F., Rosti V., Tullii G., Desii A., Tapella L., Catarsi P., Lim D., Moccia F., Antognazza M.R. (2019). Conjugated polymers optically regulate the fate of endothelial colony-forming cells. Sci. Adv..

[29] Lodola F., Vurro V., Crasto S., Di Pasquale E., Lanzani G. (2019). Optical pacing of human-induced pluripotent stem cell-derived cardiomyocytes mediated by a conjugated polymer interface. Adv. Healthc. Mater..

[30] Parameswaran R., Koehler K., Rotenberg M.Y., Burke M.J., Kim J., Jeong K.-Y., Hissa B., Paul M.D., Moreno K., Sarma N. (2019). Optical stimulation of cardiac cells with a polymer-supported silicon nanowire matrix. Proc. Natl. Acad. Sci. USA..

[31] Bruno G., Melle G., Barbaglia A., Iachetta G., Melikov R., Perrone M., Dipalo M., De Angelis F. (2021). All-optical and label-free stimulation of action potentials in neurons and cardiomyocytes by plasmonic porous metamaterials. Adv. Sci..

[32] Negri S., Faris P., Tullii G., Vismara M., Pellegata A.F., Lodola F., Guidetti G., Rosti V., Antognazza M.R., Moccia F. (2022). Conjugated polymers mediate intracellular Ca2+ signals in circulating endothelial colony forming cells through the reactive oxygen species-dependent activation of Transient Receptor Potential Vanilloid 1 (TRPV1). Cell Calcium.

[33] Gentemann L., Kalies S., Coffee M., Meyer H., Ripken T., Heisterkamp A., Zweigerdt R., Heinemann D. (2017). Modulation of cardiomyocyte activity using pulsed laser irradiated gold nanoparticles. Biomed. Opt Express.

[34] Fang J., Liu D., Xu D., Wu Q., Li H., Li Y., Hu N. (2022). Integrated Au-nanoroded biosensing and regulating platform for photothermal therapy of bradyarrhythmia. Research.

[35] Rivnay J., Owens R.M., Malliaras G.G. (2014). The rise of organic bioelectronics. Chem. Mater..

[36] Martino N., Feyen P., Porro M., Bossio C., Zucchetti E., Ghezzi D., Benfenati F., Lanzani G., Antognazza M.R. (2015). Photothermal cellular stimulation in functional bio-polymer interfaces. Sci. Rep..

[37] Ðerek V., Rand D., Migliaccio L., Hanein Y., Głowacki E.D. (2020). Untangling photofaradaic and photocapacitive effects in organic optoelectronic stimulation Devices. Front. Bioeng. Biotechnol..

[38] Manfredi G., Lodola F., Paternó G.M., Vurro V., Baldelli P., Benfenati F., Lanzani G. (2021). The physics of plasma membrane photostimulation. Apl. Mater..

[39] Gorostiza P., Isacoff E.Y. (2008). Optical switches for remote and noninvasive control of cell signaling. Science.

[40] Izquierdo-Serra M., Bautista-Barrufet A., Trapero A., Garrido-Charles A., Díaz-Tahoces A., Camarero N., Pittolo S., Valbuena S., Pérez-Jiménez A., Gay M. (2016). Optical control of endogenous receptors and cellular excitability using targeted covalent photoswitches. Nat. Commun..

[41] Leippe P., Koehler Leman J., Trauner D. (2017). Specificity and speed: tethered photopharmacology. Biochemistry.

[42] Zhang J., Wang J., Tian H. (2014). Taking orders from light: progress in photochromic bio-materials. Mater. Horiz..

[43] Fang Y., Meng L., Prominski A., Schaumann E.N., Seebald M., Tian B. (2020). Recent advances in bioelectronics chemistry. Chem. Soc. Rev..

[44] Magome N., Kanaporis G., Moisan N., Tanaka K., Agladze K. (2011). Photo-control of excitation waves in cardiomyocyte tissue culture. Tissue Eng. Part A.

[45] Riefolo F., Matera C., Garrido-Charles A., Gomila A.M.J., Sortino R., Agnetta L., Claro E., Masgrau R., Holzgrabe U., Batlle M. (2019). Optical control of cardiac function with a photoswitchable muscarinic agonist. J. Am. Chem. Soc..

[46] DiFrancesco M.L., Lodola F., Colombo E., Maragliano L., Bramini M., Paternò G.M., Baldelli P., Serra M.D., Lunelli L., Marchioretto M. (2020). Neuronal firing modulation by a membrane-targeted photoswitch. Nat. Nanotechnol..

[47] Paternò G.M., Bondelli G., Sakai V.G., Sesti V., Bertarelli C., Lanzani G. (2020). The effect of an intramembrane light-actuator on the dynamics of phospholipids in model membranes and intact cells. Langmuir.

[48] Paternò G.M., Colombo E., Vurro V., Lodola F., Cimò S., Sesti V., Molotokaite E., Bramini M., Ganzer L., Fazzi D. (2020). Membrane environment enables ultrafast isomerization of amphiphilic azobenzene. Adv. Sci..

[49] Vurro V., Bondelli G., Sesti V., Lodola F., Paternò G.M., Lanzani G., Bertarelli C. (2021). Molecular design of amphiphilic plasma membrane-targeted azobenzenes for nongenetic optical stimulation. Front. Mater..

[50] Magni A., Bondelli G., Paternò G.M., Sardar S., Sesti V. (2021). Azobenzene photoisomerization probes cell membrane nanoviscosity. arXiv.

[51] Kane C., Couch L., Terracciano C.M.N. (2015). Excitation–contraction coupling of human induced pluripotent stem cell-derived cardiomyocytes. Front. Cell Dev. Biol..

[52] Lodola F., De Giusti V.C., Maniezzi C., Martone D., Stadiotti I., Sommariva E., Maione A.S. (2021). Modeling cardiomyopathies in a dish: state-of-the-art and novel perspectives on hiPSC-derived cardiomyocytes maturation. Biology.

[53] Vurro V., Venturino I., Lanzani G. (2022). A perspective on the use of light as a driving element for bio-hybrid actuation. Appl. Phys. Lett..

[54] Raman R., Cvetkovic C., Uzel S.G.M., Platt R.J., Sengupta P., Kamm R.D., Bashir R. (2016). Optogenetic skeletal muscle-powered adaptive biological machines. Proc. Natl. Acad. Sci. USA..

[55] Park S.-J., Gazzola M., Park K.S., Park S., Di Santo V., Blevins E.L., Lind J.U., Campbell P.H., Dauth S., Capulli A.K. (2016). Phototactic guidance of a tissue-engineered soft-robotic ray. Science.

[56] Di Pasquale E., Lodola F., Miragoli M., Denegri M., Avelino-Cruz J.E., Buonocore M., Nakahama H., Portararo P., Bloise R., Napolitano C. (2013). CaMKII inhibition rectifies arrhythmic phenotype in a patient-specific model of catecholaminergic polymorphic ventricular tachycardia. Cell Death Dis..

[57] Lodola F., Morone D., Denegri M., Bongianino R., Nakahama H., Rutigliano L., Gosetti R., Rizzo G., Vollero A., Buonocore M. (2016). Adeno-associated virus-mediated CASQ2 delivery rescues phenotypic alterations in a patient-specific model of recessive catecholaminergic polymorphic ventricular tachycardia. Cell Death Dis..

[58] Salvarani N., Crasto S., Miragoli M., Bertero A., Paulis M., Kunderfranco P., Serio S., Forni A., Lucarelli C., Dal Ferro M. (2019). The K219T-Lamin mutation induces conduction defects through epigenetic inhibition of SCN5A in human cardiac laminopathy. Nat. Commun..

[59] Lian X., Zhang J., Azarin S.M., Zhu K., Hazeltine L.B., Bao X., Hsiao C., Kamp T.J., Palecek S.P. (2013). Directed cardiomyocyte differentiation from human pluripotent stem cells by modulating Wnt/β-catenin signaling under fully defined conditions. Nat. Protoc..

[60] Nakahama H., Di Pasquale E. (2016). Generation of cardiomyocytes from pluripotent stem cells. Methods Mol. Biol..

[61] Vurro V., Scaccabarozzi A.D., Lodola F., Storti F., Marangi F., Ross A.M., Paternò G.M., Scotognella F., Criante L., Caironi M., Lanzani G. (2020). A polymer blend substrate for skeletal muscle cells alignment and photostimulation. Adv. Photonics Res..

[62] Lucas, B.D., and Kanade, T. An Iterative Image Registration Technique with an Application to Stereo Vision. In IJCAI'81: 7th international joint conference on Artificial intelligence,2 674-679

[63] Shi J., Tomasi (1994). In Proceedings of IEEE Conference on Computer Vision and Pattern Recognition CVPR-94.

