# Supplementary material for: Optical modulation of excitation-contraction coupling in human-induced pluripotent stem cell-derived cardiomyocytes
Source: iScience. 2023 Feb 2;26(3):106121. doi: 10.1016/j.isci.2023.106121 (PMC9984557; doi:10.1016/j.isci.2023.106121)
Supplement: Document S1. Figures S1–S5 [file mmc1.pdf]

iScience, Volume *26*

Supplemental information

Optical modulation of excitation-contraction coupling in human-induced pluripotent stem cell-derived cardiomyocytes

Vito Vurro, Beatrice Federici, Carlotta Ronchi, Chiara Florindi, Valentina Sesti, Silvia Crasto, Claudia Maniezzi, Camilla Galli, Maria Rosa Antognazza, Chiara Bertarelli, Elisa Di Pasquale, Guglielmo Lanzani, and Francesco Lodola

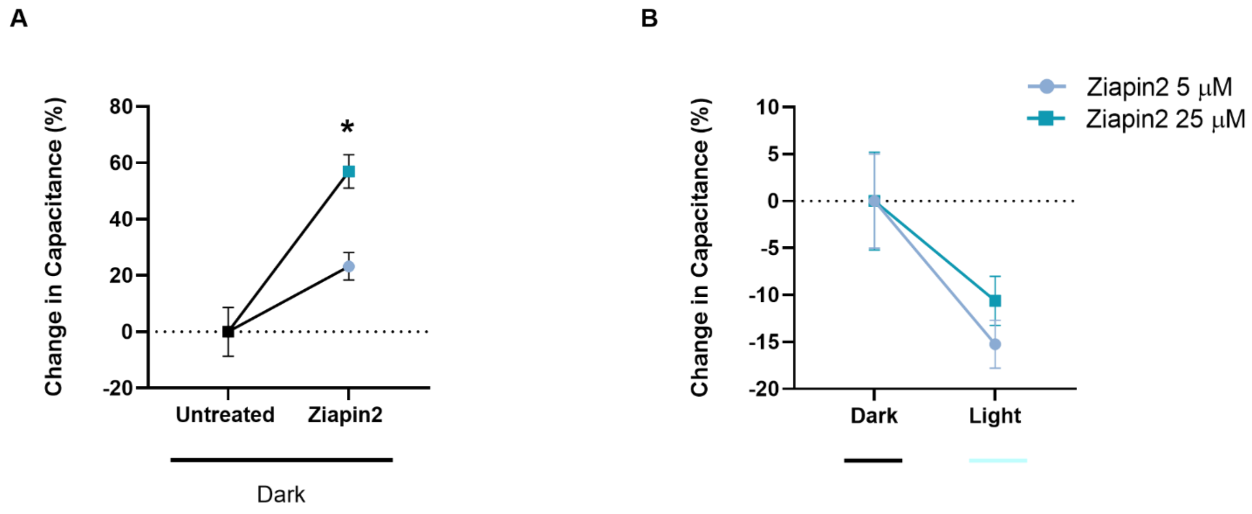

Figure S1. Ziapin2-mediated modulation of membrane capacitance. Evaluation of capacitance changes in hiPSC-CMs upon Ziapin2 membrane partitioning (**panel A**) and photoisomerization (**panel B**). Ziapin2 5μM represented with light blue dots while 25 μM in teal squares. Light power density, 80 mW/mm^2. The experiments were carried out at room temperature (24°C). n = 15 and 20 for untreated and Ziapin2-loaded cells respectively from three independent differentiations. Data are represented as mean ± SEM.

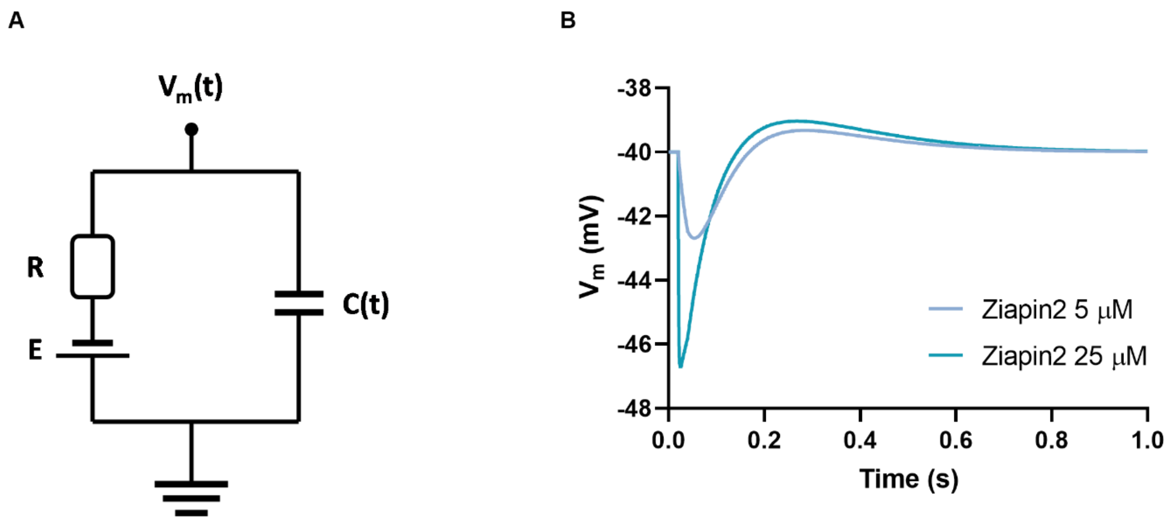

Figure S2. Simulation of membrane voltage. Simulated equivalent circuit (**panel A**) and capacitance-induced membrane potential variation (**panel B**). This simulation, performed as previously described,[46] mimics the effect of a 20 ms light pulse on Ziapin2 photoisomerization (a capacitance variation) and subsequent membrane potential modulation. To reproduce the observed effect, only the photoisomerization characteristic time has been reduced (t_{hyp} = 35 ms for 5 mM and t_{hyp} = 1 ms for 25 mM). This could be consistent with the localization of Ziapin2 in membrane domains more prone to favour isomerization.

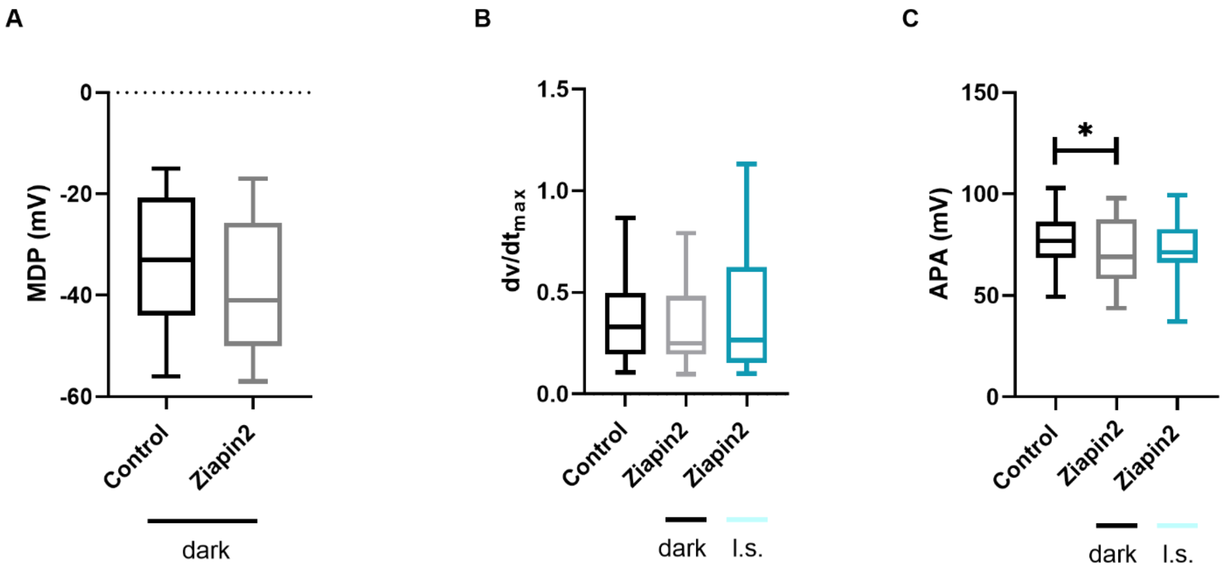

Figure S3. AP parameters in hiPSC-CMs exposed to Ziapin2. Comparison of the maximum diastolic potential (MDP, **panel A**), maximal rate of rise (dv/dt_{max}, **panel B**) and AP amplitude (APA, **panel C**) between spontaneous and light-evoked APs in 25µM Ziapin2 loaded hiPSC-CMs ($n > 40$ for each condition from three independent differentiations). Data are presented as box and whiskers plot (min to max). Light power density, 80 mW/mm^2. The experiments were carried out at room temperature (24°C). * $p < 0.05$.

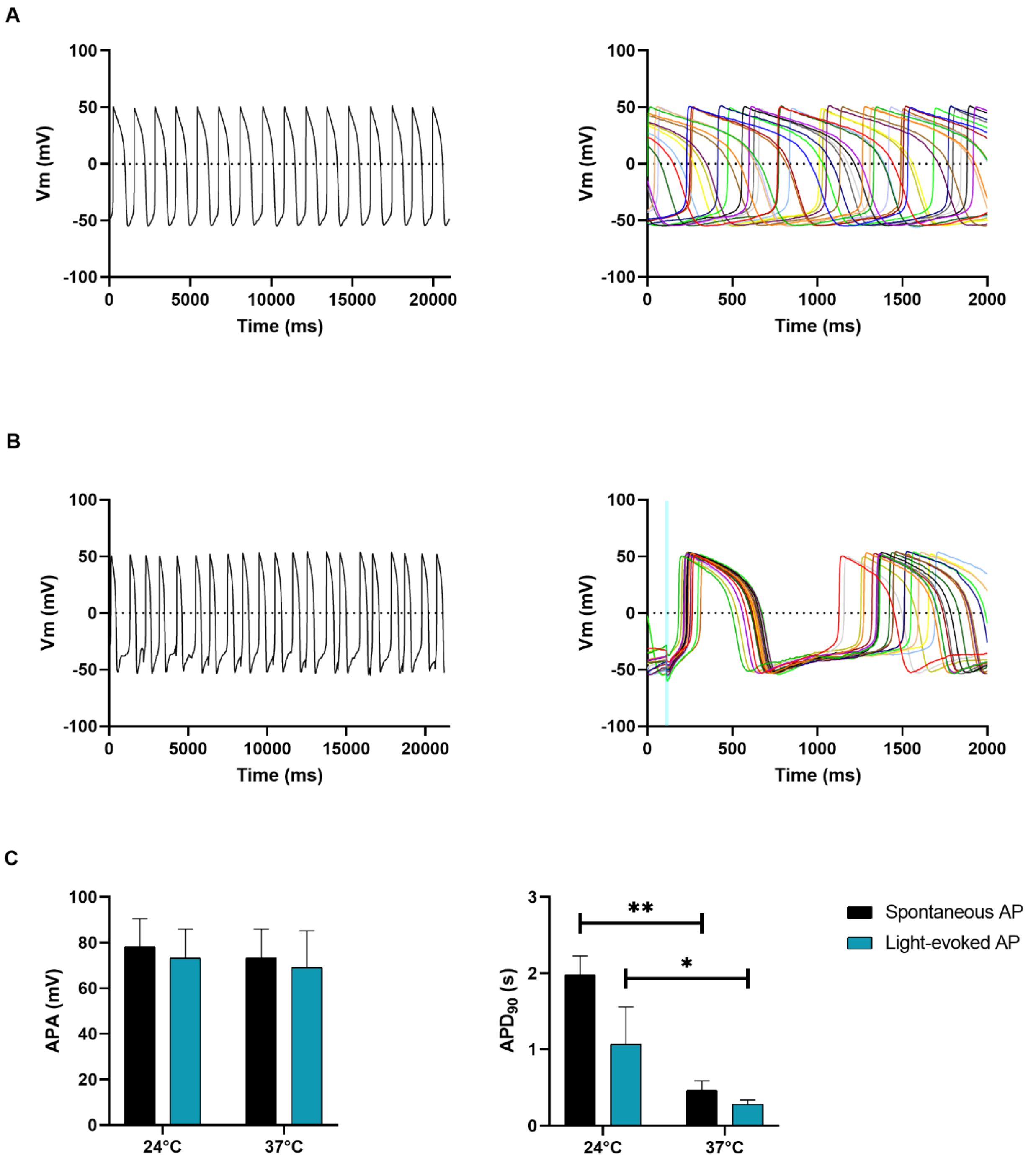

Figure S4. Light-induced Vm modulation in Ziapin2 loaded hiPSC-CMs at 37°C. A) Representative trace of spontaneous APs (left) and their distribution over a 2 second timescale (right). **B)** Representative trace of light-evoked APs (left) and their distribution over a 2 second timescale (right). The APs were generated in response to a light train stimulation (20 ms cycle length; 0.5 Hz stimulation frequency, represented as cyan shaded areas). Light power density, 80 mW/mm^2. The experiments were carried out at 37°C. **C)** Comparison between spontaneous and light-evoked AP parameters (i.e., AP amplitude, APA and AP duration at 90% of repolarization, APD_{90}) recorded either at room temperature or at 37°C ($5<n<30$, data are shown as mean ± SEM). * $p < 0.05$, ** $p < 0.01$.

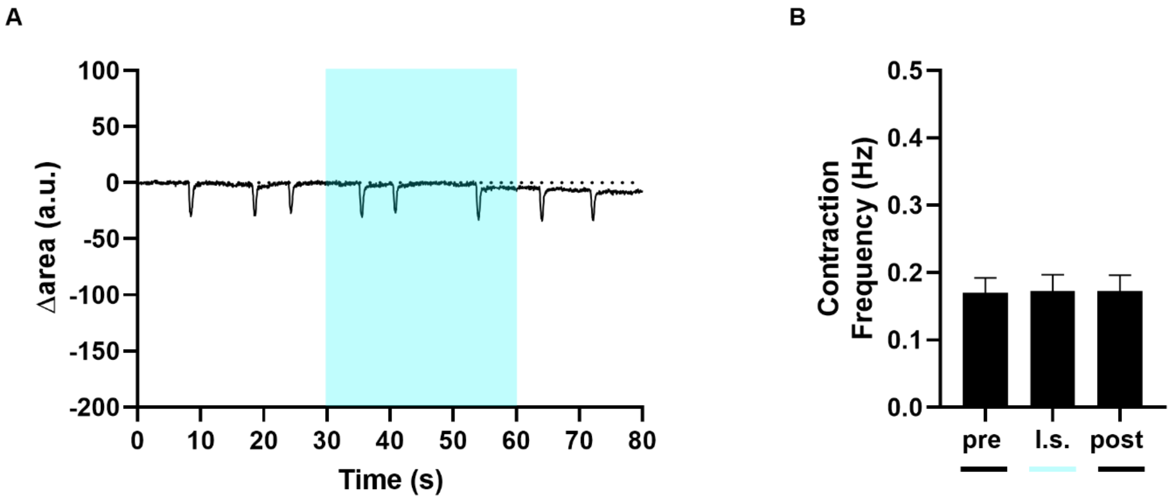

Figure S5. Light-induced contraction rate modulation in hiPSC-CMs incubated with vehicle. A) Representative trace of the contraction behaviour of DMSO-treated hiPSC-CMs. **B)** Contraction frequency before (pre), during (l.s.), and after (post) 1 Hz pulsed light stimulation at light power density = 30 mW/mm^2 (n = 25 from three independent differentiations, data are shown as mean ± SEM). The experiments were carried out at room temperature (24°C).
